# Development of Liposomal and Liquid Crystalline Lipidic Nanoparticles with Non-Ionic Surfactants for Quercetin Incorporation

**DOI:** 10.3390/ma16165509

**Published:** 2023-08-08

**Authors:** Ioannis Tsichlis, Athanasia-Paraskevi Manou, Vasiliki Manolopoulou, Konstantina Matskou, Maria Chountoulesi, Vasiliki Pletsa, Aristotelis Xenakis, Costas Demetzos

**Affiliations:** 1 Section of Pharmaceutical Technology, Department of Pharmacy, School of Health Sciences, National and Kapodistrian University of Athens, Panepistimioupolis Zografou, 15771 Athens, Greece; gtsichlis@pharm.uoa.gr (I.T.); athanasia.manou@gmail.com (A.-P.M.); vanessamanwl@gmail.com (V.M.); demetzos@pharm.uoa.gr (C.D.); 2 Institute of Chemical Biology, National Hellenic Research Foundation, 48 Vassileos Constantinou Avenue, 11635 Athens, Greece; kmatskou@eie.gr (K.M.); vpletsa@eie.gr (V.P.); arisx@eie.gr (A.X.)

**Keywords:** liposomes, lyotropic liquid crystals, lipid nanoparticles (LNPs), drug delivery nanosystems, quercetin, stealth

## Abstract

The aim of the present study is the development, physicochemical characterization, and in vitro cytotoxicity evaluation of both empty and quercetin-loaded HSPC (hydrogenated soy phosphatidylcholine) liposomes, GMO (glyceryl monooleate) liquid crystalline nanoparticles, and PHYT (phytantriol) liquid crystalline nanoparticles. Specifically, HSPC phospholipids were mixed with different non-ionic surfactant molecules (Tween 80 and/or Span 80) for liposomal formulations, whereas both GMO and PHYT lipids were mixed with Span 80 and Tween 80 as alternative stabilizers, as well as with Poloxamer P407 in different ratios for liquid crystalline formulations. Subsequently, their physicochemical properties, such as size, size distribution, and ζ-potential were assessed by the dynamic and electrophoretic light scattering (DLS/ELS) techniques in both aqueous and biological medium with serum proteins. The in vitro biological evaluation of the empty nanosystems was performed by using the MTT cell viability and proliferation assay. Finally, the entrapment efficiency of quercetin was calculated and the differences between the two different categories of lipidic nanoparticles were highlighted. According to the results, the incorporation of the non-ionic surfactants yields a successful stabilization and physicochemical stability of both liposomal and liquid crystalline nanoparticles. Moreover, in combination with an appropriate biosafety in vitro profile, increased encapsulation efficiency of quercetin was achieved. Overall, the addition of surfactants improved the nanosystem’s stealth properties. In conclusion, the results indicate that the physicochemical properties were strictly affected by the formulation parameters, such as the type of surfactant.

## 1. Introduction

Quercetin is a natural flavonoid that has been widely studied due to its antioxidant, anti-inflammatory, antitumoral, and antiviral properties. However, some limitations make quercetin delivery extremely challenging, including poor bioavailability, low aqueous solubility, and low stability [1]. Therefore, several perspectives have been developed to overcome these problems. Among them, novel lipid drug delivery nanosystems have gained increasing attention as they present various advantages in delivering both hydrophilic and hydrophobic molecules, like quercetin [2].

Liposomes and liquid crystalline nanoparticles are used as nanocarriers in drug delivery and other biomedical applications. Specifically, liposomes are spherical vesicles composed of lipid bilayers, which resemble the structure of cell membranes. They can encapsulate both hydrophilic and hydrophobic molecules within their aqueous core and lipid bilayers, respectively. They present several advantages as drug delivery vehicles including biocompatibility, targeted drug delivery, controlled drug release, and enhanced stability. However, they exhibit some limitations, such as low drug loading, and recognition by the immune system, which leads to reduced circulation time [3]. Contrariwise, liquid crystalline nanoparticles are self-assembled nanostructures formed by amphiphilic molecules that arrange into liquid crystalline phases. These nanoparticles can encapsulate drugs within their organized structures and possess a unique nanoscale ordering. Among their advantages, they present high drug loading, enhanced stability, tunable drug release profile based on their internal nanostructures, and enhanced permeability through the biological barriers [4].

Liposomes are widely used to improve quercetin oral absorption and targeting, increase its antioxidant activity, and ensure sustained release [5]. There are several formulations that have been proven to be effective considering the delivery of quercetin. It was discovered that the laccase- and quercetin-containing liposomes enhanced the release of quercetin, as well as of laccase at an acidic pH, and induced the death of tumor cells [6]. Moreover, the biochemical/pathological abnormalities in diabetic nephropathy were found to be improved by the liposomal form of quercetin. Additionally, the liposomal form resulted in a higher concentration of quercetin in plasma [7]. In another study, liposomes coated with Eudragit-containing quercetin were formulated for intestine delivery. A variety of structures from small, spherical, uni-, and bi-lamellar to multi-lamellar liposomes were observed. The human intestinal cells were highly protected against oxidative stress by lowering the generation of reactive oxygen species and incorporating quercetin into the vesicles [8].

Sterically stabilized liposomes (stealth) are also called long-circulating liposomes as they can escape phagocytosis and remain in the blood for a long time. In terms of their structure, they are usually small in size and present attached polymers on their surface, such as polyethylene glycol (PEG) and polyvinyl alcohol, which offer a kind of shield [9]. These modifications of the liposomal surface restrict the interactions with macrophage cells (stereochemical or steric stabilization), making them invisible from the mononuclear phagocyte system, which is an essential prerequisite for achieving long circulation time [10].

When amphiphilic molecules consisting of a hydrophilic head group connected with a hydrophobic hydrocarbon chain region are dispersed in water, they self-assemble into different mesophases, which are termed as lyotropic liquid crystalline systems. This self-assembly process takes place to reduce the system’s free energy by placing the hydrophilic part towards the aqueous environment, and as a result, the hydrophobic parts remain in the interior, minimizing the interaction with water. The most well-known mesophases, because of their stability characteristics, are the lamellar ones (liposomes), the hexagonal (hexosomes) ones, and the bicontinuous cubic phases (cubosomes) [11]. Specifically, the bicontinuous cubic phase consists of a 3D network, splitting two distinct, continuous but nonintersecting, hydrophilic sections. As regards the inverse hexagonal phase, it concerns a 2D structure that is composed of water-filled cylindrical rods (hydrophilic nanochannels) embedded in a continuous hydrophobic medium. The aforementioned mesophases, cubosomes, and hexosomes are categorized as non-lamellar nanoparticles, and it is worth mentioning that they can be used as drug delivery nanosystems [12]. The lipids that have proven to be the most useful for the configuration of cubic and hexagonal phases are glyceryl monooleate (GMO, 2,3-dihydroxypropyl oleate) and phytantriol (PHYT, 3,7,11,15-tetramethyl-1,2,3-hexadecanetriol). However, the addition of a stabilizer is necessary to receive stable colloidal dispersions. Therefore, Pluronic^®^ polymers and especially Pluronic^®^ F-127 (Poloxamer P407) are widely used as stabilizers [13,14].

Span 80 (sorbitan monooleate) and Tween 80 (polyoxyethylene sorbitan monooleate) are non-ionic surfactants that are allowed and widely used in food, cosmetics, and pharmaceutical industries since they exhibit less toxicity, higher biocompatibility, and biodegradability [15]. Different surfactant combinations of Span and Tween have been successfully used in formulating emulsions and microemulsions [16,17]. Span 80 is a lipophilic surfactant and has a hydrophilic–lipophilic balance (HLB) value of 4.3, whereas Tween 80 is a hydrophilic surfactant that has an HLB value of 15 [18]. The HLB value influences the size of the nanoparticle and defines the significant technological properties of a system. Span and Tween are used as HLB regulators in biphasic formulations [19]. Therefore, lyotropic liquid crystal gels were prepared using mixtures of Span 80 and Tween 80 [20]. Although there are literature examples of non-ionic surfactants [21,22,23,24] or lipid nanocarriers [25,26,27,28] used for quercetin encapsulation, knowledge regarding the incorporation of quercetin in hybrid lipid nanosystems mixed with non-ionic surfactants has not been mentioned before, at least to the best of the authors’ knowledge.

The aim of the present study is the development and physicochemical characterization of both empty and quercetin-loaded HSPC (hydrogenated soy phosphatidylcholine) liposomes, GMO (glyceryl monooleate) liquid crystalline nanoparticles, and PHYT (phytantriol) liquid crystalline nanoparticles. Moreover, in vitro biological evaluation of the empty nanosystems was performed, by using the MTT assay, to assess the inhibition of cell proliferation and cytotoxicity in the WM164 melanoma cell line. More specifically, liposomes were modified with Tween 80 and Span 80 at different ratios, whereas liquid crystalline nanoparticles were stabilized by Tween 80 or/and Span 80 as alternative stabilizers, as well as with P407 in different ratios. Dynamic and electrophoretic light scattering techniques were utilized to determine the size, size distribution, and ζ-potential of the prepared nanosystems.

## 2. Materials and Methods

### 2.1. Materials

L-α-phosphatidylcholine hydrogenated (soy) (HSPC) was purchased from Avanti Polar Lipids Inc. (Alabaster, AL, USA) and used without further purification. Surfactant Tween 80 was obtained from Syndesmos S.A. (Athens, Greece), while Span 80 was procured from Sigma-Aldrich Chemical Co. (St. Louis, MO, USA). The chloroform, methanol, and H_2_O used were of HPLC grade and purchased from Thermo Fisher Scientific Inc. (Waltham, MA, USA). In addition, fetal bovine serum (FBS) was obtained from Thermo Fischer Scientific Inc. (Waltham, MA, USA). Quercetin was purchased from Fluka, BioChemika (Buchs, Switzerland). The lipids used for the preparation of the liquid crystalline nanosystems were glyceryl monooleate Monomuls^®^ 90-O18 [GMO, 1-(cis-9-octadecenoyl)-rac-glycerol], which was purchased from BASF (Ludwigshafen, Germany), and phytantriol (PHYT, 3,7,11,15-tetramethyl-1,2,3-hexadecanetriol), which was purchased from DSM Nutritional Products Ltd. (Heerlen, The Netherlands). Both lipids were used without further purification. As regards the stabilizer, Pluronic^®^ F-127 (Poloxamer P407) (PEO_98_-PPO_67_-PEO_98_), with an average molar mass of 12,600 g/mol, was purchased from Sigma-Aldrich Chemical Co. (St. Louis, MO, USA).

### 2.2. Methods

#### 2.2.1. Preparation of Liposomal Systems

A thin-film hydration method was performed for the preparation of all liposomal systems investigated in this study. Appropriate amounts of HSPC mixed with a surfactant (Span 80 or/and Tween 80) were dissolved in chloroform and then transferred into a round bottom flask, which was connected to a rotary evaporator (Rota vapor R-114, Buchi, Switzerland). The vacuum was applied, and the thin film was formed in the wall of the flask by slow evaporation of the solvent at 40 °C for 30 min. Subsequently, it was hydrated with HPLC-grade water to a final lipid concentration of 10 mg/mL by slow stirring for 1 h in a water bath above the phase transition temperature of HSPC (60 °C). The resultant multi-lamellar vesicles (MLVs) were subjected to two sonication cycles (amplitude 70, cycle 0.7) of 5 min interrupted by a 5 min resting period using a probe sonicator (UP 200S, dr. hielsher GmbH, Berlin, Germany). The resultant small uni-lamellar vesicles (SUVs) were allowed to anneal for 30 min [29,30]. In the case of quercetin-loaded liposomes, the quercetin was dissolved with the lipid in chloroform to obtain the formation of the film.

In this study, eight liposomal systems were prepared by the thin-film hydration method. The empty liposomes included HSPC, HSPC:Span 80 (9:1 molar ratio), HSPC:Tween 80 (9:1 molar ratio), and HSPC:Span 80:Tween 80 (9:0.5:0.5 molar ratio). These systems were also utilized to incorporate quercetin. Specifically, these systems included HSPC:quercetin (9:0.1 molar ratio), HSPC:Span 80:quercetin (9:1:0.1 molar ratio), HSPC:Tween 80:quercetin (9:1:0.1 molar ratio), and HSPC:Tween 80:Span 80:quercetin (9:0.5:0.5:0.1 molar ratio).

#### 2.2.2. Preparation of Liquid Crystalline Nanoparticles

All liquid crystalline systems were prepared by the usage of the top–down method (TD). Particularly, the lipid phase which consisted of GMO or PHYT and the surfactant Span 80 were weighted into glass vials and heated to 60 °C until free flowing. Thereinafter, the appropriate volume of HPLC-grade water solution (pH = 6.0), which included different amounts of the stabilizer P407 and/or the surfactant Tween 80, was added to the vials containing the lipids to achieve a lipid concentration of 20 mg/mL. The mixtures were first sonicated using a bath sonicator for 2 min at 60 °C. Then, they were subjected to two 2 min sonication cycles (amplitude 70, cycle 0.7) and interrupted by a 2 min resting period, using a probe sonicator (UP 200 S, DrHielsher GmbH, Berlin, Germany), until a milky dispersion was formed. The received dispersions were allowed to anneal for 30 min and then stored at room temperature. In the cases of liquid crystalline systems with entrapped quercetin, the appropriate amounts of GMO or PHYT and quercetin were fully dissolved in ethanol. The ethanol was gently evaporated until a dry film of the lipid–quercetin mixture was achieved. The mixture was heated to 60 °C until free-flowing. Afterward, we repeated the same process described above for the empty liquid crystalline nanosystems. The concentration of quercetin in the final dispersion was 2 mg/mL [31].

Specifically, in this study, liquid crystalline systems were prepared by the method that is described above. The liquid crystalline systems that included GMO were as follows: GMO:P407 (4:1 weight ratio), GMO:Span 80:P407 (4:1:0.5 weight ratio), GMO:Tween 80 (4:1 weight ratio), and GMO:Span 80:Tween 80:P407 (4:0.5:0.5:0.5 weight ratio). These systems were also utilized to incorporate quercetin. All systems containing quercetin were prepared at a lipid/quercetin 10:1 ratio. Furthermore, the liquid crystalline systems that included PHYT were as follows: PHYT:P407 (4:1 weight ratio), PHYT:Span 80:P407 (4:0.5:0.5 weight ratio), PHYT:Span 80 (4:1 weight ratio), PHYT:Span 80:Tween 80:P407 (4:0.5:0.5:0.5 weight ratio), and PHYT:Tween 80 (weight ratio 4:1). These systems were also utilized to incorporate quercetin at a lipid/quercetin 10:1 ratio.

#### 2.2.3. Dynamic and Electrophoretic Light Scattering

The physicochemical characteristics of nanosystems were evaluated by measuring their mean hydrodynamic diameter (D_h_), polydispersity index (PDI), and ζ-potential. Specifically, the mean hydrodynamic diameter and the polydispersity index (PDI) were measured by dynamic light scattering (DLS), while the ζ-potential was measured by electrophoretic light scattering (ELS). These studies were performed by diluting 100 μL of each sample with 2900 μL HPLC-grade water. Measurements were performed at a detection angle of 90°, at 25 °C, in a photon correlation spectrometer (Zetasizer 3000 HSA, Malvern, UK) and analyzed by the CONTIN method (Zetasizer 3000 HSA, MALVERN software). The colloidal stability of the nanosystems was studied over time in order to investigate the effect of the non-ionic surfactant molecules and the triblock copolymer P407.

#### 2.2.4. Incubation in Fetal Bovine Serum

Following their preparation, liposomes and liquid crystalline nanoparticles were dispersed in FBS (t = 1 day). These studies were performed by diluting 100 μL of liposomal/liquid crystalline dispersion with 2900 μL of fetal bovine serum. Finally, nanosystems were incubated at room temperature for 15 min and light scattering measurements were carried out in the biological medium. The physicochemical properties of the empty nanosystems were assessed in fetal bovine serum (FBS) as they are directly correlated with the type of proteins they interact with and the degree of protein uptake. Hence, this protocol could inform us about the existence of stealth properties in our nanosystems [32,33].

#### 2.2.5. Entrapment Efficiency Determination

The free quercetin was separated from the quercetin entrapped in nanosystems by using the ultrafiltration centrifugal method. Specifically, the dispersion was centrifuged for 45 min at 8000× *g* rpm using centrifugal filter tubes (molecular weight (MW) cutoff = 10 kDa; Merck Millipore, Darmstadt, Germany) at 4 °C. The nanoparticles were separated from the aqueous phase, and the free quercetin was analyzed in the supernatant. The UV-Vis spectrophotometry method was used to measure the free drug concentration in the samples after centrifugation and supernatant collection. The absorption measurements were performed at a wavelength of 372 nm by using a UV-Vis spectrophotometer (Shimadzu PharmaSpec UV-1700 UV-Vis spectrophotometer, Shimadzu Corporation, Kyoto, Japan) and a pre-constructed calibration curve. Centrifuged samples of the respective empty nanosystems were used as a blank. The entrapment efficiency (*EE*) % was calculated according to the following equation:

$$\left(EE\right)\%=\left(1-\frac{C_{supernatant}}{C_{total}}\right)\%$$

where *C_supernatant_* is the quercetin concentration that was quantified in the supernatant (non-entrapped) and *C_total_* is the total concentration of the quercetin added in the dispersion [34].

#### 2.2.6. Cell Culture and Cell Proliferation Assay

The human melanoma cell line WM164 (BRAFV600E, p53Y220C; Wistar Institute Melanoma Research Centre, https://wistar.org/) was generously provided by Dr. G. Skretas (National Hellenic Research Foundation, Athens, Greece). The cells were grown in Dulbecco’s Modified Eagle’s Medium (DMEM) containing 4.5 g/L glucose, L-glutamine, and pyruvate, supplemented with 10% FBS and 1% penicillin/streptomycin (Gibco-Life Technologies, Grand Island, NY, USA), at 37 °C in a humidified incubator with 5% CO_2_ [35,36].

The cytotoxicity of nanocarriers in vitro was assessed 72 h after treatment by MTT assay. The cells were added to 96-well plates at a density of 12 × 10^3^ cells/well and incubated for 24 h at 37 °C under 5% CO_2_. On the day of administration, supernatants from the wells were aspirated and replaced with fresh growth medium containing different concentrations of the developed liposomes from 0.01 mg/mL to 0.5 mg/mL. MTT solution (0.05 mg/100 μL) was added to each well and incubated for 3 h. At the end of the incubation time, the medium was removed and the insoluble purple formazan crystals were diluted in isopropanol [29]. The absorbance of the converted dye was measured at a wavelength of 570 nm and was used to determine cell viability as following:

$$\mathrm{Cell}\;\mathrm{viability}\;(\%)=\frac{OD\;of\;treated\;cells}{OD\;of\;control}\times 100$$

where *OD* is the optical density.

## 3. Results and Discussion

### 3.1. Physicochemical Characterization of the Prepared Nanoparticles

The physicochemical properties of the prepared nanosystems, including the mean hydrodynamic diameter (D_h_), the polydispersity index (PDI), and the ζ-potential, were characterized. Specifically, the mean hydrodynamic diameter (D_h_) and the polydispersity index (PDI) of the nanoparticles were determined by dynamic light scattering (DLS), while the ζ-potential was measured by electrophoretic light scattering (ELS). The stabilizing efficiency results of each stabilizer, either alone or in combinations, are presented in Table 1, and the corresponding physicochemical results of the systems are presented in Table 2 and refer to the day of liposome preparation (t = 0 days). Data represent the mean values and standard deviation of three replicates of each sample.

**Figure 1:**
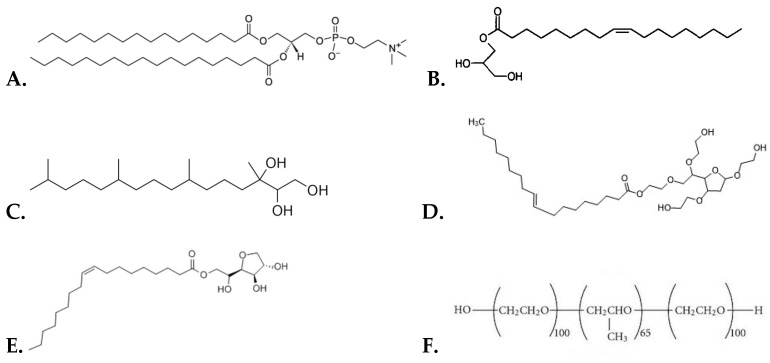
The chemical structures of (**A**) HSPC lipid, (**B**) GMO lipid, (**C**) PHYT lipid, (**D**) Tween 80, (**E**) Span 80, and (**F**) Poloxamer P407.

According to Table 2, the D_h_ value of HSPC liposomes was greater compared to HSPC:Span 80 and HSPC:Tween 80 nanosystems on the day of their preparation. On the other hand, liposomes composed of HSPC:Span 80:Tween 80 had a higher D_h_ value concerning the pure HSPC liposomes. The GMO:Tween 80 nanosystem showed the lowest D_h_ value of all the systems on the day of their preparation. GMO:P407 and GMO:Span 80:Tween 80:P407 nanosystems had similar and slightly higher D_h_ values compared to GMO:Tween 80, while GMO:Span 80:P407 had the highest value. These findings are consistent with the literature, where it is mentioned that increasing P407 concentration results in the formation of smaller nanoparticles due to the reduction of the interfacial tension between lipid and water, which leads to a larger surface area [37]. This is also explained by the classical emulsification theory and the fact that P407, as a hydrophilic polymer, increases the positive curvature of cubosomes, whereas the hydrophobic lipid increases the negative curvature [38,39]. All chemical structures of the used materials are illustrated in Figure 1. Also, we noticed that the D_h_ values of PHYT lipid formulations are higher than the respective GMO ones. This size difference observed for the different lipids used is due to the fact that there are differences in the degree of interaction and the principal mode of stabilization of GMO- and PHYT-based dispersions by P407. It is worth mentioning that the PPO block of P407 has a lower affinity for the phytantriol bilayer, resulting in simple adsorption of P407 to the particle surface when compared to GMO [38].

Regarding PDI, we observed that all liposomal nanosystems composed of HSPC/surfactant had a smaller PDI value compared to pure HSPC liposomes. HSPC:Tween 80 liposomes showed a smaller PDI value concerning the other empty liposomes, and as a result, this nanosystem is characterized by greater homogeneity. Moreover, the GMO:Span 80:P407 nanosystem had the highest value. GMO:P407 and GMO:Tween 80 nanosystems had similar PDI values, with GMO:Tween 80 having the lowest; therefore, it is characterized by the greatest homogeneity among the four systems. Concerning the systems that were prepared with the PHYT lipid, apart from the reference system (PHYT:P407), the system composed of PHYT:Tween 80 yielded the lowest PDI value. Therefore, it can be characterized as the most homogeneous system. Generally, Span 80 has a hydrophilic-lipophilic balance (HLB) value of 4.3, whereas Tween 80 has an HLB value of 15 [18]. The HLB value of Tween 80 is the most advantageous feature that can be attributed to the system’s enhanced ability to stabilize the dispersions [40]. Since both surfactants have the same hydrophobic chain, the higher efficiency of Tween 80 may be due to the presence of the hydrophilic chains, which may produce a higher free energy of adsorption [41].

The ζ-potential of the empty liposomal nanosystems was found to be below 30 mV. The small values of ζ-potential indicate that the electrostatic repulsive forces are not strong enough to overcome the attractive van der Waals forces and prevent agglomeration between suspended colloidal particles. However, these small values of ζ-potential were predictable and could be attributed to the components of the above liposomes. In particular, the neutrally charged HSPC lipid and the surfactants used for the liposome preparation are not ionized in the presence of an aqueous medium, thus maintaining ζ-potential values below 30 mV. This might consequently affect the physical stability of the nanosystems during their storage [42]. The ζ-potential values of all the prepared GMO nanosystems were highly negative, as was expected according to other research studies [24,38,43]. As for the nanoparticles prepared with PHYT and P407, they presented high negative values of ζ-potential, and this is probably because of the fact that the PEO block of P407 covered the external surface of the nanoparticles and absorbed hydroxyl anions from the aqueous medium [34]. As we observed, the addition of the surfactants either did not affect the value of the ζ-potential or increased it and made it more negative, which is a fact that reduced the tendency for flocculation. An exception was the system PHYT:Span which had an elevated positive ζ-potential value.

According to Table 3, the mean hydrodynamic diameter was increased in all liposomal nanosystems after the incorporation of quercetin. This fact can be attributed to the development of van der Waals interactions between the lipid bilayer and quercetin. Specifically, a smaller D_h_ value was observed in HSPC:Span 80:quercetin liposomes. On the other hand, the HSPC:quercetin liposomal nanosystem had a higher D_h_ value compared to the other nanosystems, which was about 1.2 μm.

After the incorporation of quercetin, the nanosystem with the smallest PDI value was HSPC:Span 80:Tween 80:quercetin. Therefore, this nanosystem exhibits greater homogeneity compared to the other quercetin-loaded liposomes.

For the GMO-prepared nanosystems, after the incorporation of quercetin, the GMO:P407:quercetin had the lowest value of the mean hydrodynamic diameter, with a quite low PDI value; therefore, it is considered to be the most homogeneous nanosystem. Compared to empty nanosystems, we observed various changes among them. The GMO:P407:quercetin and the GMO:Span 80:P407:quercetin had decreased D_h_ values, the GMO:Tween 80:quercetin had an increased D_h_ value, while the GMO:Span 80:Tween:P407:quercetin system had almost the same D_h_ value as the empty one. Concerning PDI, the GMO:P407:quercetin and the GMO:Tween 80:quercetin had increased PDI values, while the GMO:Span 80:P407:quercetin and the GMO:Span 80:Tween 80:P407:quercetin had decreased PDI values.

We also noticed that D_h_ values of PHYT lipid formulations with entrapped quercetin were relatively low; however, they were quite comparable with those of the reference system.

As mentioned above, for the polydispersity index value, the lower the price the more effective and homogenous the system. Considering the PDI results of the nanosystems that were prepared with PHYT, it is obvious that there is a difference between the stability offered by Span 80 and Tween 80. Nanosystems with Span 80 also needed P407 to be stabilized, as opposed to Tween 80, which was capable of stabilizing the systems on its own. 

### 3.2. Colloidal Stability of the Nanosystems over Time

**Figure 2:**
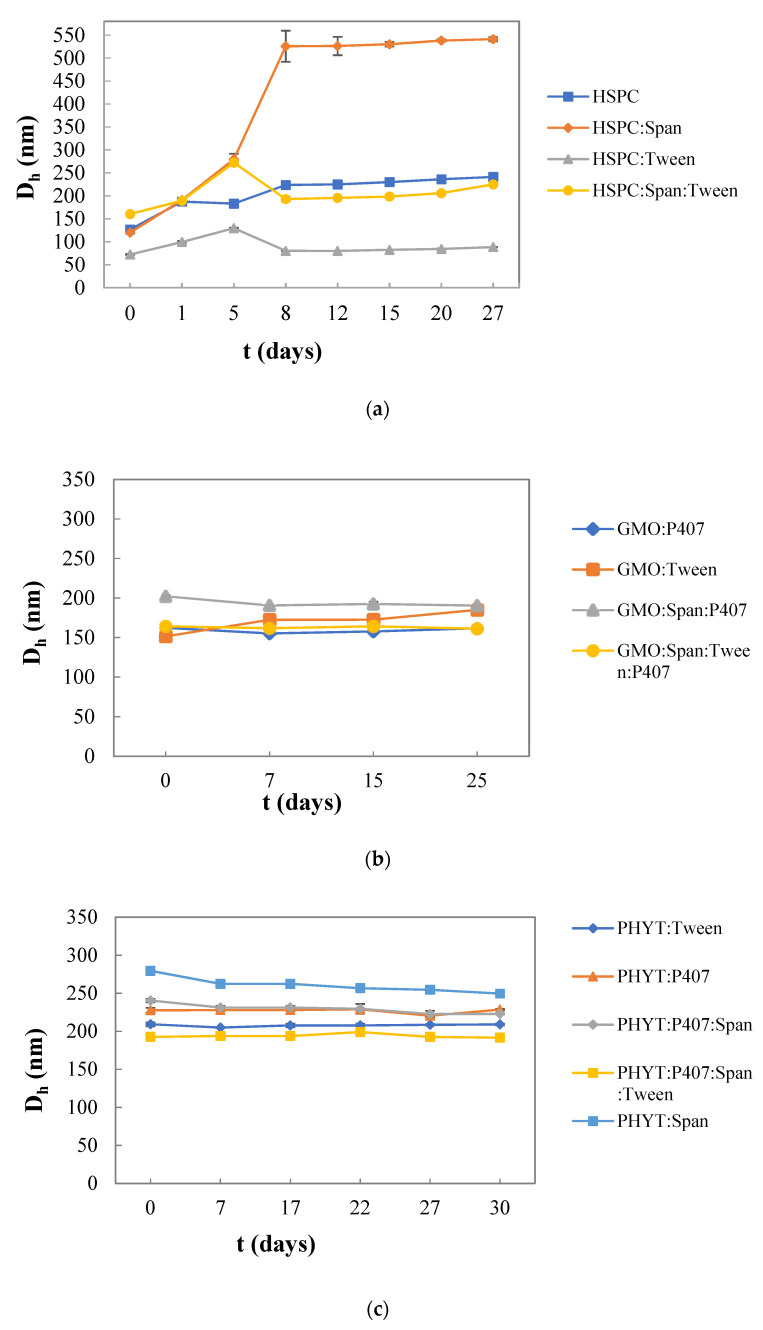
Stability assessment of the size (D_h_, nm) of (**a**) HSPC liposomes, (**b**) GMO liquid crystalline nanoparticles, and (**c**) PHYT liquid crystalline nanoparticles over time.

As it is shown in Figure 2a, the HSPC:Tween 80 liposomal nanosystem had a smaller mean hydrodynamic diameter compared to the other empty nanosystems, being quite stable during the 27 days. On the other hand, a higher mean hydrodynamic diameter was observed in HSPC:Span 80 liposomes 5 days after their preparation. Similar results have been also obtained from other research studies, which suggest that the inclusion of surfactants within lipid-based vesicles has an obvious effect on vesicle size. The decrease in the particle size depends on the HLB value and it might be observed with an increase in the HLB value of the surfactant. Specifically, in this study, Tween 80 (HLB value 15), which is more hydrophilic than Span 80 (HLB value 4.3), increased the repulsive hydration forces, resulting in the formation of liposomes with a mean hydrodynamic diameter smaller than 130 nm [42,44].

As regards the liquid crystalline nanoparticles and in particular the GMO-based one, all the dispersions were proven to be stable over time, while no significant changes were observed to the D_h_ values during the 25 days. More specifically, the GMO:Span 80:P407 system presents the highest mean hydrodynamic diameter, compared to the other empty nanosystems, presenting a small decrease on the 7th day. However, after the 7th day, no significant change in D_h_ values was noted. On the other hand, GMO:Tween 80 increased over days, but still did not exceed 30 nm. Generally, P407 is responsible for the colloidal stability of the particles since it can provide an efficient steric stabilization of the liquid crystalline mesophases in colloidal dispersions of nanoparticles. The hydrophobic part is attracted to the surface of the particle or strongly linked in the lipid bilayers, while the hydrophilic chains (PEO) extend outward into the aqueous environment. Therefore, the PEO block sprawls out of the particle’s surface, preventing the aggregation of the particles and enhancing the stability of the systems [45,46]. Regarding the mean hydrodynamic diameter values of the systems prepared with PHYT lipids, small differences were observed from day to day, apart from the PHYT:Span 80 nanosystem, whose changes were slightly more noticeable, showing a gradual decrease from day 7th day onwards. Moreover, this was the system with the highest D_h_ values, while the system that yielded the lowest D_h_ values was PHYT:P407:Span 80:Tween 80, which showed a small increase on the 22nd day but then returned. The system PHYT:P407:Span 80 fluctuated, but was generally stable. The system PHYT:Tween 80 was very stable, with very small to non-existent fluctuations. Apart from the PHYT:Span 80 system which stood out a bit, the rest of the systems showed similar D_h_ values. In conclusion, the size of all systems during those 30 days remained stable.

Regarding PDI for the systems that were prepared with GMO, in general, they appeared to be quite homogenous. GMO:Tween 80 and GMO:Span 80:Tween 80:P407 obtained the most stable values over time, while GMO:Span 80:P407 had a noticeable decrease on the 7th day and GMO:P407 an increase on the 25th day (Figure S1b). Concerning the systems prepared with a PHYT lipid, a variation among the PDI values of the systems was detected. More specifically, the liquid crystalline system PHYT:Tween 80 presented a PDI value around 0.2, which is comparable to those of the system PHYT:P407, presenting a decrease on the 7th, and then it stabilized. The specific value showed satisfactory size uniformity. Meanwhile, PHYT:P407:Span 80 system had a PDI value slightly higher than 0.3. PHYT:Span 80, and PHYT:P407:Span 80:Tween 80 liquid crystalline systems showed PDI values higher than 0.4, revealing reduced uniformity and a higher tendency towards flocculation and aggregation [29]. The PHYT:P407:Span 80 system presented a small decrease on the 7th and 22nd day, but in the end, it returned. The system PHYT:P407:Span 80:Tween 80 presented fluctuations, but in the end, it returned close to the initial value. In all liquid crystalline systems, small fluctuations occurred over time, but no difference between the maximum and minimum values exceeded 0.1. It follows that the PDI of all systems remained stable during the stability study (Figure S1c).

We should mention that the heads of Tween 80 do not cover the surface but penetrate the core. The cluster looks like a cell, in the case of Tween 80, where the walls are built of PEO groups and the tunnels are filled with water. They create a network hidden inside the particle. Also, the Tween 80 creates a network with dispersed hydrogen bonds relative to the Span 80, due to its longer heads. The Span creates a dense network of hydrogen bonds in the core and, more sparsely, at the surface. In conclusion, Tween 80 works better alone as a stabilizer than Span 80; however, when they are used together, there is a good cooperativity between them, yielding to efficient stabilization [15].

### 3.3. The Effect of the Serum Proteins on the Physicochemical Behavior of the Prepared Liposomal Nanosystems

**Figure 3:**
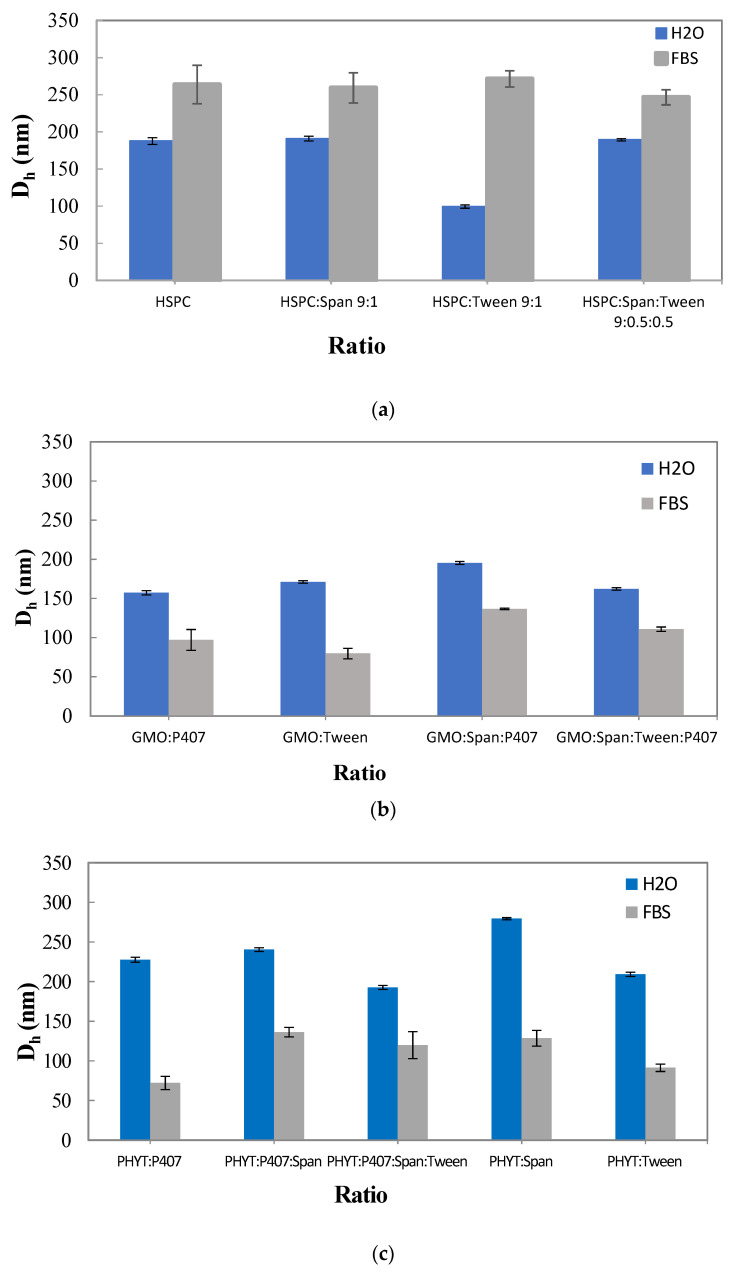
Hydrodynamic diameter (D_h_, nm) of (**a**) HSPC liposomes, (**b**) GMO liquid crystalline nanoparticles, and (**c**) PHYT liquid crystalline nanoparticles in aqueous and biological medium (FBS).

The physicochemical properties of the empty nanosystems were characterized after incubation in FBS one day after their preparation (t = 1 day) (Figure 3).

The mean hydrodynamic diameter of the empty liposomal nanosystems was significantly increased after incubation in FBS. Specifically, a higher D_h_ value was observed in the HSPC:Tween 80 liposomal nanosystem, while a smaller D_h_ value was measured in HSPC:Span 80:Tween 80. However, even in the presence of proteins, the size remains lower than 280 nm, indicating good stability, especially in the case of HSPC:Span 80:Tween 80. The D_h_ of the GMO systems was significantly decreased after dilution and incubation with FBS. This is described in the previous literature as a result of the use of P407 and the fact that the polymeric corona is not able to be protected against the albumin contained in FBS [34]. Cubosomes interact with the amphiphilic albumin that breaks up cubosomes solubilizing the GMO lipid [47]. D_h_ values of systems prepared with PHYT lipid were decreased almost to half value. The size decrease in FBS of all the studied nanosystems prepared with PHYT lipid takes place, according to Azmi et al., because a structural transition from the biphasic phase (Pn3m cubic coexisting with a hexagonal (HII) phase) to a neat hexagonal (HII) phase is observed [11].

The polydispersity index of the empty liposomal nanosystems as well as both the liquid crystalline nanosystems had the maximum value after incubation in FBS, indicating that there was great heterogeneity, due to the formation of supramolecular aggregates of the initial nanostructures with proteins and the other serum components. The high inhomogeneity (high values of PDI) is also related to the many different components of the FBS (albumin and other serum proteins).

### 3.4. In Vitro Cytotoxicity Studies

**Figure 4:**
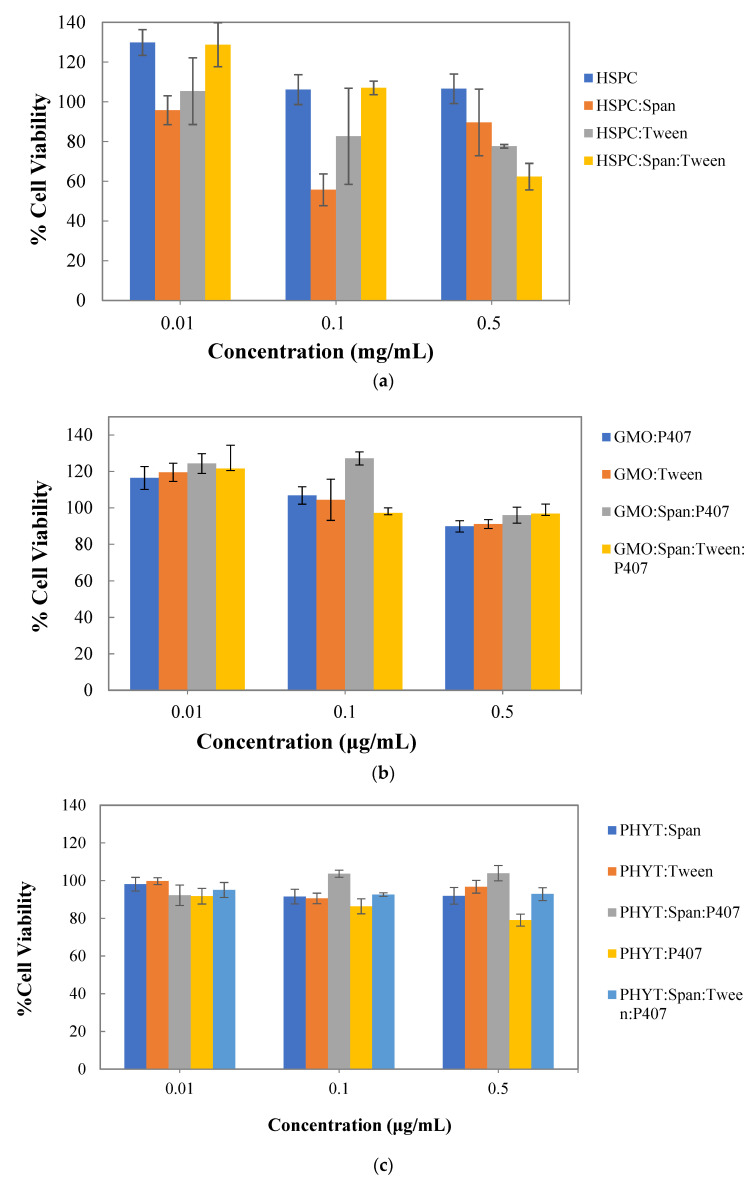
In vitro cell viability results of (**a**) HSPC liposomes, (**b**) GMO liquid crystalline nanoparticles, and (**c**) PHYT liquid crystalline nanoparticles in WM164 cells after 72 h of treatment.

A fundamental step in determining the safety of a bioactive molecule or a nanocarrier in eukaryotic cells is the use of cell viability assays. In general, cell viability assays are used to determine the concentration range or a specific concentration of the nanocarrier, which can be used to exploit its properties in further research studies [35]. In this study, the MTT assay was used to assess the cytotoxicity of the developed liposomes in the WM164 melanoma cell line. Therefore, WM164 cells were treated, for 72 h, with various concentrations of the empty nanosystems to clearly define the dosages for further in vitro experiments. The results are presented in Figure 4.

Liposomes composed of pure HSPC lipids were well tolerated during a 72 h period by the WM164 cells at a concentration range between 0.01 mg/mL and 0.5 mg/mL in the culture medium. Regarding HSPC:Tween 80 liposomes, cell viability results showed that this nanosystem was well tolerated by the WM164 cells at 0.01 mg/mL; however, an approximately 20% decrease in cell viability was observed at higher concentrations. On the other hand, HSPC:Span 80 liposomes, although well tolerated at 0.01 mg/mL, exhibited 56% cell viability at 0.1 mg/mL concentration, indicating that this nanosystem may have a potent cytotoxic effect on WM164 cells. Moreover, HSPC:Span 80:Tween 80 liposomes, also well tolerated at the concentrations of 0.01 mg/mL and 0.1 mg/mL, significantly inhibited cell proliferation (40% decrease in cell viability) at a concentration of 0.5 mg/mL. Overall, HSPC lipid nanoparticles were not cytotoxic at the concentration range used, which was between 0.01 mg/mL and 0.5 mg/mL.

All GMO lipid nanosystems exhibited no toxicity to the WM164 cells after a 72 h period at a concentration range between 0.01 μg/mL and 0.5 μg/mL in the culture medium. It is worth noting that in all cases, GMO lipid nanosystems consistently enhance cell proliferation at 0.01 μg/mL, which is in agreement with previous results, where a significant increase in cell viability with no changes in cell cycle, probably due to a positive effect of GMO on cell proliferation, was observed in a rat pheochromocytoma PC12 cell line at low GMO concentrations [48].

The PHYT liquid crystalline nanoparticles, in general, exhibited no cytotoxicity compared to the control samples, even at the highest tested concentration of 0.5 µg/mL, indicating their potential as safe carriers, at least for the concentration range studied.

Overall, the cell viability assay revealed that liposomes composed of HSPC mixed with different surfactant molecules resulted in a greater decrease in cell viability compared to pure HSPC liposomes. This might be explained by the fact that surfactants can effectively solubilize cell membranes and, thus, lead to cell lysis. On the other hand, the GMO and PHYT liquid crystalline nanosystems were proven to be non-toxic at the examined concentrations and, therefore, are suitable for further investigation as successful and safe drug delivery systems.

### 3.5. Quercetin Entrapment Results

**Figure 5:**
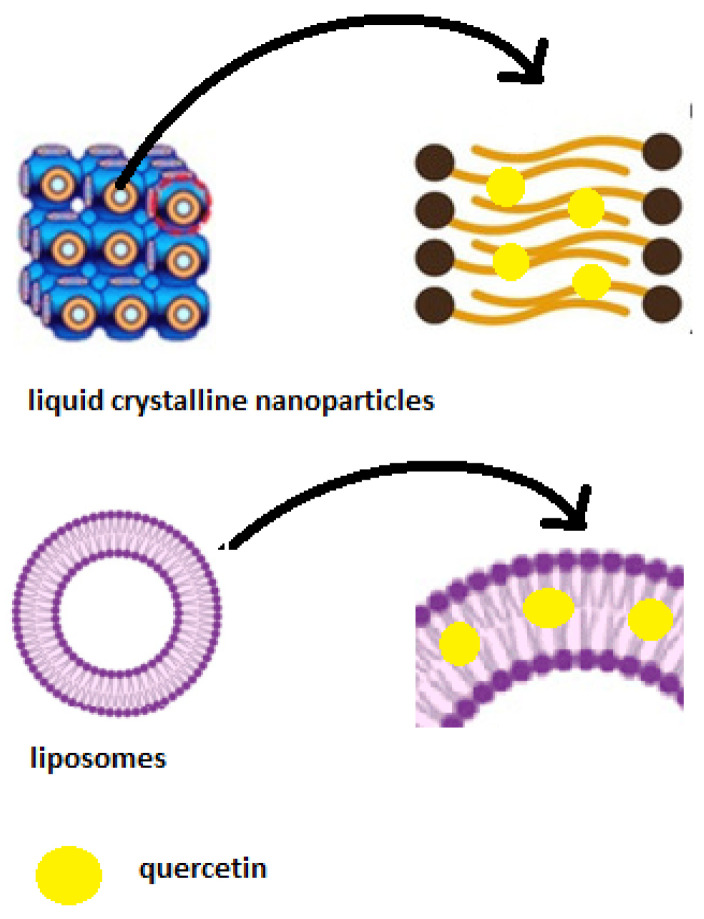
Schematic representation of the prepared nanosystems with incorporated quercetin. Adapted by [50,51].

The entrapment efficiency (EE) values of quercetin are presented in Table 3, while the schematic representation of the quercetin incorporation is illustrated in Figure 5. It seemed that the addition of a surfactant to different lipid-based nanosystems had little effect on the EE of quercetin, as the values were all higher than 80%. Moreover, the entrapment efficiency of the liquid crystalline nanosystems was enhanced compared to the entrapment efficiency of the liposomes.

The received results showed that quercetin entrapment efficiency was above 80% for every one of the liquid crystalline systems that were prepared with PHYT. Making the comparison between the reference system and the systems with Span 80 and Tween 80, the entrapment efficiency value also shows that the use of a combination of surfactants can increase the entrapment efficiency, where the entrapment efficiency of the systems, especially with the Span 80, exhibit the highest entrapment efficiency, which may be due to the fact that quercetin is a hydrophobic molecule. These results follow the results of research conducted by Lv et al., where the results obtained indicate that the combination of Tween–Span non-ionic surfactants will increase entrapment efficiency [49]. Furthermore, as we can observe, Tween 80 is not capable of providing increased entrapment efficiency by itself, in contrast to Span 80.

As for the physicochemical characterization of quercetin-loaded liposomes, the D_h_ value of HSPC:quercetin liposomes was extremely high during the period of 27 days. Similar results were obtained from the liposomes composed of HSPC:Span 80:quercetin. On the other hand, liposomes composed of HSPC:Tween 80:quercetin and HSPC:Span 80:Tween 80:quercetin were found to be stable in terms of particle size, with a relatively small mean hydrodynamic diameter (approximately 180 nm). Thus, the presence of the surfactants Tween 80 or a combination of Tween 80 and Span 80 exhibited a good effect, while they improved the stability profile of the liposomes loaded with quercetin, which is probably due to the formation of a hydrophilic external corona that prevented the liposomal aggregation and disruption.

All the systems that were prepared with GMO and contained quercetin presented great stability over time. The D_h_ values of all the nanosystems had a relatively small mean hydrodynamic diameter (approximately 175 nm) and almost no difference over the 21 days. GMO:P407:quercetin had the lowest values of D_h_, while GMO:Tween 80:quercetin had the highest. The combination of the stabilizer P407 and the surfactants Span 80 and Tween 80 seems to be very efficient.

**Figure 6:**
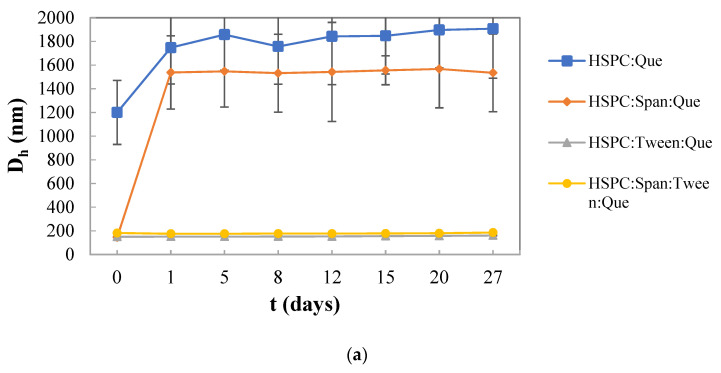

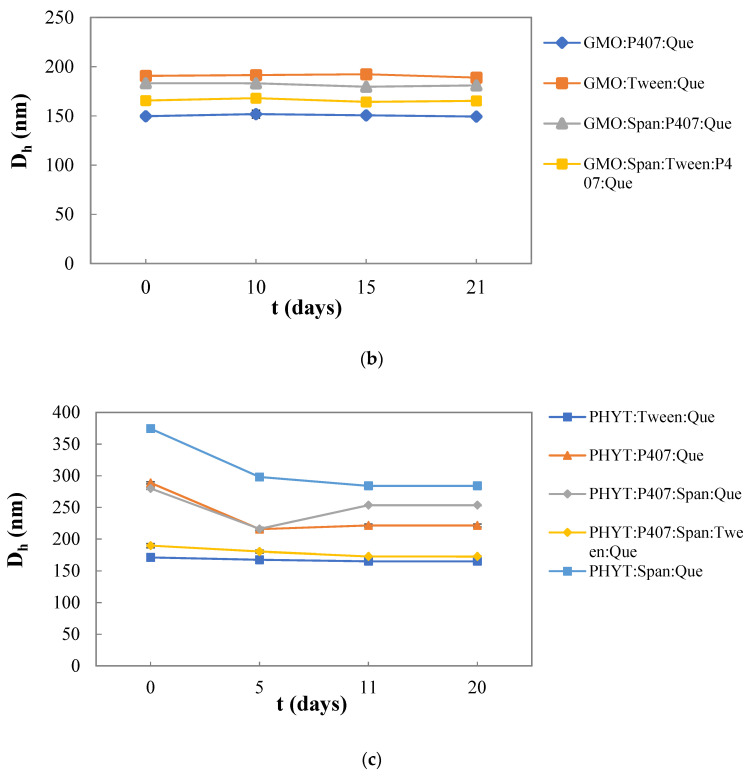
Stability assessment of the size (D_h_, nm) of (**a**) HSPC liposomes, (**b**) GMO liquid crystalline nanoparticles, and (**c**) PHYT liquid crystalline nanoparticles with entrapped quercetin over time.

All liquid crystalline systems prepared with PHYT were characterized in terms of physicochemical stability over a period of 30 days of storage at room temperature. On the day of their preparation, we could observe (Figure 6c) differences in D_h_ values among the systems. The nanosystems composed of PHYT showed a satisfactory physicochemical stability profile, with their sizes decreasing up to 150 nm during the stability study. The system PHYT:Span 80:quercetin had the highest D_h_ values, presenting a decrease on the 5th day and stabilization. The most stable systems seem to be PHYT:Tween 80:quercetin and PHYT:P407:Span 80:Tween 80:quercetin. The system PHYT:P407:Span 80:quercetin presented a decrease on the 5th day, and then it returned to the initial value. We derive the conclusion that Tween is capable of stabilizing the liquid crystalline systems, whereas Span does not have this capability. This happens because Tween 80 predominates the lipophilic part, interacting more strongly with the lipid bilayer by entering the channels of the liquid crystals, and therefore the system is stabilized [52]. On the other hand, Span 80 turns the polar heads towards the aqueous part, leaving freedom of movement in the liquid crystal channels. Therefore, we observe a lack of stability. However, Span 80 can be used in combination with Tween 80, providing similar physicochemical characteristics to Tween 80 alone and high entrapment efficiencies of quercetin.

Concerning the PDI, all the systems have significantly similar and low values; therefore, they are characterized by great homogeneity. The PDI results follow the D_h_ results, confirming great stability over time for all four nanosystems prepared with GMO lipid. Regarding the systems loaded with quercetin, PHYT:Span 80:quercetin exhibited the highest PDI values. The surface-charged drug loaded liquid crystalline systems, PHYT:Tween 80:quercetin, PHYT:P407:Span 80:Tween 80:quercetin, and PHYT:P407:Span 80:quercetin, presented smaller PDI values, indicating more heterogeneous systems. Apart from the system PHYT:P407:quercetin, whose value becomes smaller with time, all the other systems seem to be stable over time. As we infer from Figure 6c, concerning the reference system PHYT:P407:quercetin, non-ionic surfactants stabilize the quercetin-loaded systems more efficiently.

## 4. Conclusions

According to the literature, the liquid crystalline nanoparticles and the liposomes exhibit promising potential in the horizons of lipid-based drug delivery systems to deliver small molecules as well as macromolecules of hydrophilic and lipophilic nature, like quercetin. According to Singh et al. [14], the liquid crystalline nanoparticles loaded with insulin and quercetin demonstrated significantly higher plasma C_max_ values in comparison with quercetin suspension and a physical mixture of quercetin and insulin. In addition, targeting the brain, transdermal administration, and antioxidant activity are some of the properties of quercetin that are enhanced when quercetin is encapsulated in liposomes.

In the present study, regarding the physicochemical characterization of the developed liposomes, we observed that the addition of a surfactant in the liposomal nanosystem resulted in the reduction of size. The HSPC:Tween 80 liposomal nanosystem had a smaller mean hydrodynamic diameter compared to the other empty nanosystems, being more stable during the 27 days. The liquid crystalline nanoparticles and in particular the GMO-based dispersions were proven to be stable over time, while no significant changes were observed to the D_h_ values. In terms of the mean hydrodynamic diameter, the systems PHYT:Tween 80 and PHYT:P407:Span 80:Tween 80 seem to be the most stable.

On the other hand, both HSPC:Tween 80:quercetin and HSPC:Span 80:Tween 80:quercetin were found to be more stable in terms of particle size, with relatively small mean hydrodynamic diameters. Thus, the presence of the surfactant Tween 80 or a combination of Tween 80 and Span 80 improved the stability profile of the liposomes loaded with quercetin. All the systems that were prepared with GMO and contained quercetin presented great stability over time. Furthermore, the nanosystems PHYT:Tween 80:quercetin and PHYT:P407:Span 80:Tween 80:quercetin were found to be more stable in comparison with the reference system. The combination of the stabilizer P407 and the surfactants Span 80 and Tween 80 seems to be very efficient, improving the stability profile of the liquid crystalline nanoparticles loaded with quercetin.

The ζ-potential values of all liposomal nanosystems were below 30 mV; however, the agglomeration between suspended colloidal particles was prevented by the surfactant corona covering the liposomes. The ζ-potential values of the prepared GMO nanosystems were highly negative, as expected according to other research studies, probably due to the van der Waals attractions, impurities, and the PEO group of P407. As for the nanoparticles prepared with PHYT, they also presented highly negative ζ-potential values except for the nanosystem PHYT:Span, which had an elevated positive ζ-potential value.

After incubation in fetal bovine serum (FBS), the empty liposomes exhibited an increase in the hydrodynamic diameter. However, even in the presence of the proteins the size remained lower than 280 nm, indicating good stability, especially in the case of HSPC:Span 80:Tween 80 liposomes. On the other hand, the D_h_ values of both GMO and PHYT nanosystems were significantly decreased after incubation in FBS. Moreover, the MTT assay revealed that liposomes composed of HSPC mixed with different surfactant molecules resulted in a statistically significant decrease in cell viability compared to pure HSPC liposomes. Contrariwise, the GMO and PHYT liquid crystalline nanosystems were proven to be non-toxic at the examined concentrations and can be considered as suitable for further investigation as successful and safe drug delivery systems.

In conclusion, the presence of the non-ionic surfactants enhanced the physicochemical stability of the resultant lipidic nanoparticles, both liposomal and liquid crystalline ones. Moreover, the increased encapsulation efficiency of quercetin that was achieved in combination with their proper biosafety in vitro profile enhances their high potential as quercetin delivery nanosystems.

## Figures and Tables

**Table 1:** The stabilizing efficiency of the proposed stabilizer depending on the lipid.

	HSPC	GMO	PHYT
Span 80	✓	x	x
Tween 80	✓	✓	✓
Span 80:Tween 80	✓	x	x
Span 80:P407	-	✓	✓
Tween 80:P407	-	-	-
Span 80:Tween 80:P407	-	✓	✓

✓: Efficiently stabilized with homogenous dispersion; x: not efficiently stabilized with non-dispersed aggregates.

**Table 2:** The physicochemical characteristics of the empty nanosystems in HPLC-grade water, t = 0 days. Results are the mean ± standard deviation.

Sample	Ratio	D_h_ (nm)	PDI	ζ-Potential (mV)
HSPC	-	126.6 ± 0.4	0.380 ± 0.008	−19.6 ± 10.7
HSPC:Span 80	9:1	119.9 ± 2.1	0.345 ± 0.007	−14.9 ± 2.4
HSPC:Tween 80	9:1	72.4 ± 0.8	0.261 ± 0.010	−3.8 ± 1.3
HSPC:Span 80:Tween 80	9:0.5:0.5	160.5 ± 0.4	0.320 ± 0.022	−5.6 ± 1.2
PHYT:P407	4:1	227.6 ± 3.1	0.283 ± 0.003	−28.9 ± 5.3
PHYT:P407:Span 80	4:0.5:0.5	240.4 ± 2.3	0.379 ± 0.021	−29.9 ± 9.5
PHYT:P407:Span 80:Tween 80	4:0.5:0.5:0.5	192.6 ± 2.5	0.434 ± 0.031	−4.9 ± 13.0
PHYT:Span 80	4:1	279.4 ± 1.3	0.413 ± 0.024	15.1 ± 16.0
PHYT:Tween 80	4:1	209.2 ± 2.6	0.234 ± 0.015	−32.8 ± 0.4
GMO:P407	4:1	162.3 ± 1.0	0.214 ± 0.025	−22.1 ± 0.4
GMO:Tween 80	4:1	151.5 ± 0.4	0.212 ± 0.013	−26.6 ± 9.0
GMO:Span 80:P407	4:1:0.5	202.2 ± 1.4	0.461 ± 0.026	−37.3 ± 0.8
GMO:Span 80:Tween 80:P407	4:0.5:0.5:0.5	164.2 ± 0.6	0.327 ± 0.003	−28.4 ± 1.0

**Table 3:** The physicochemical characteristics and the entrapment efficiency of the quercetin-loaded nanosystems in HPLC-grade water, t = 0 days. Results are the mean ± standard deviation.

Sample	Ratio	D_h_ (nm)	PDI	Entrapment Efficiency of Quercetin (%)
HSPC:Quercetin	9:0.1	1200.3 ± 270.4	1.000 ± 0.000	86
HSPC:Span 80:Quercetin	9:1:0.1	144.0 ± 1.2	0.307 ± 0.013	84
HSPC:Tween 80:Quercetin	9:1:0.1	149.9 ± 0.5	0.353 ± 0.020	83
HSPC:Span 80:Tween 80:Quercetin	9:0.5:0.5:0.1	181.5 ± 3.7	0.298 ± 0.005	83
PHYT:P407: Quercetin	4:1	288.8 ± 1.7	0.562 ± 0.147	80
PHYT:P407:Span 80:Quercetin	4:0.5:0.5	279.9 ± 1.4	0.233 ± 0.137	85
PHYT:P407:Span 80:Tween 80:Quercetin	4:0.5:0.5:0.5	189.6 ± 3.3	0.261 ± 0.002	86
PHYT:Span 80:Quercetin	4:1	374.4 ± 4.5	0.455 ± 0.130	86
PHYT:Tween 80:Quercetin	4:1	171.2 ± 2.6	0.278 ± 0.009	82
GMO:P407:Quercetin	4:1	149.7 ± 2.5	0.272 ± 0.005	97
GMO:Tween 80:Quercetin	4:1	190.6 ± 1.8	0.272 ± 0.010	98
GMO:Span 80:P407:Quercetin	4:1:0.5	183.2 ± 1.0	0.307 ± 0.036	97
GMO:Span 80:Tween 80:P407:Quercetin	4:0.5:0.5:0.5	165.6 ± 0.2	0.265 ± 0.014	99

## Data Availability

Not applicable.

## Supplementary Materials

The following supporting information can be downloaded at https://www.mdpi.com/article/10.3390/ma16165509/s1. Figure S1: Stability assessment of the polydispersity index (PDI) of (a). HSPC liposomes, (b). GMO liquid crystalline nanoparticles, and (c). PHYT liquid crystalline nanoparticles over time. Figure S2: Polydispersity index (PDI) of (a). HSPC liposomes, (b). GMO liquid crystalline nanoparticles, and (c). PHYT liquid crystalline nanoparticles in aqueous and biological medium (FBS). Figure S3: Stability assessment of the polydispersity index (PDI) of (a). HSPC liposomes, (b). GMO liquid crystalline nanoparticles, and (c). PHYT liquid crystalline nanoparticles with entrapped quercetin over time.
