# Supplementary material for: Development of Liposomal and Liquid Crystalline Lipidic Nanoparticles with Non-Ionic Surfactants for Quercetin Incorporation
Source: Materials (Basel). 2023 Aug 8;16(16):5509. doi: 10.3390/ma16165509 (PMC10456281; doi:10.3390/ma16165509)
Supplement: Supplementary file 1 [file materials-16-05509-s001.zip › materials-2487248-supplementary.pdf]

Supporting Information

Development of Liposomal and Liquid Crystalline Lipidic Nanoparticles with Non-Ionic Surfactants for Quercetin Incorporation

Ioannis Tsichlis [1,†], Athanasia-Paraskevi Manou [1,†], Vasiliki Manolopoulou [1,†], Konstantina Matskou [2], Maria Chountoulesi [1,*], Vasiliki Pletsa [2], Aristotelis Xenakis [2] and Costas Demetzos [1]

[1] Section of Pharmaceutical Technology, Department of Pharmacy, School of Health Sciences, National and Kapodistrian University of Athens, Panepistimioupolis Zografou, 15771 Athens, Greece; gtsichlis@pharm.uoa.gr (I.T.); athanasia.manou@gmail.com (A.-P.M.); vanessamanwl@gmail.com (V.M.); demetzos@pharm.uoa.gr (C.D.)

[2] Institute of Chemical Biology, National Hellenic Research Foundation, 48 Vassileos Constantinou Avenue, 11635 Athens, Greece; kmatskou@eie.gr (K.M.); vpletsa@eie.gr (V.P.); arisx@eie.gr (A.X.)

* Correspondence: mchountoules@pharm.uoa.gr

† These authors contributed equally to this work.

a.

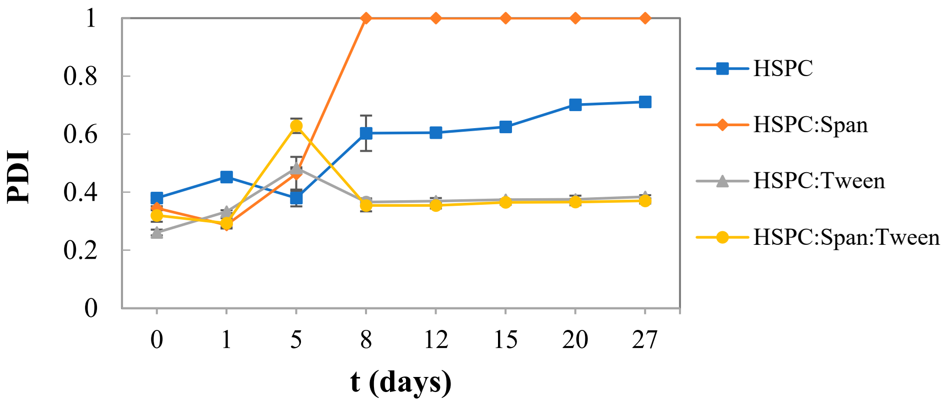

b.

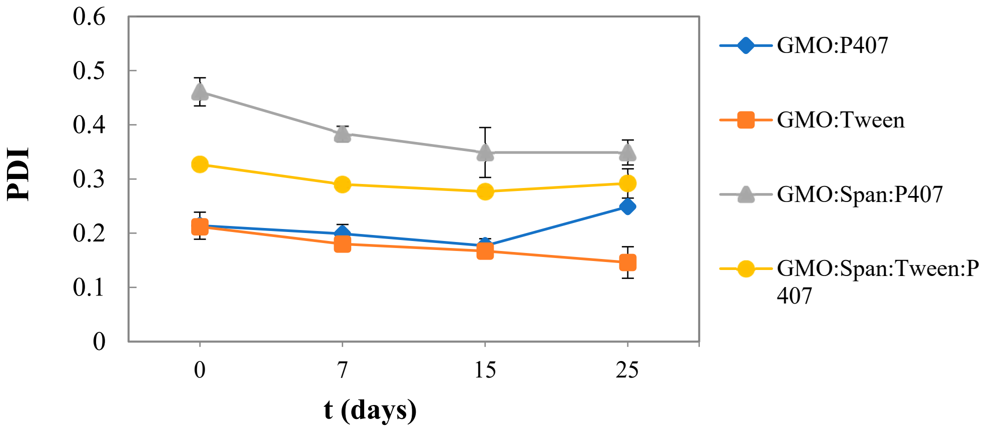

c.

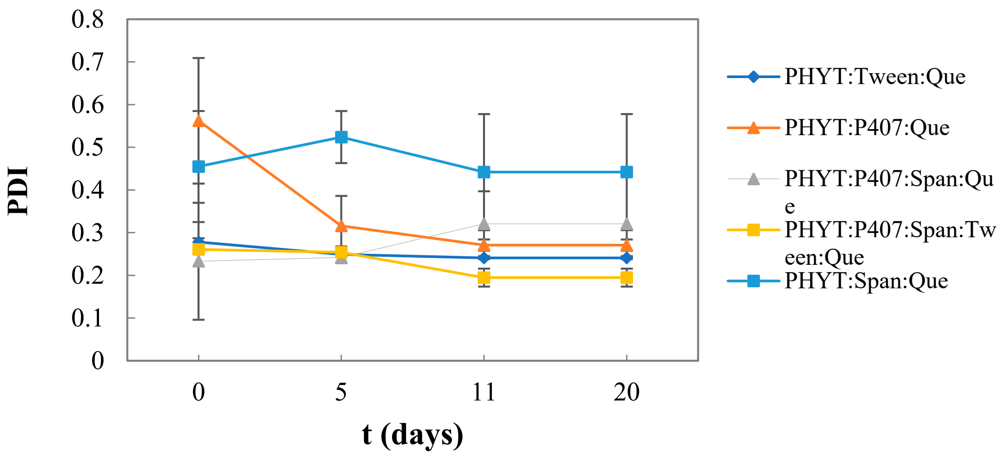

Figure S1. Stability assessment of the polydispersity index (PDI) of **a.** HSPC liposomes, **b.** GMO liquid crystalline nanoparticles and **c.** PHYT liquid crystalline nanoparticles over time.

a.

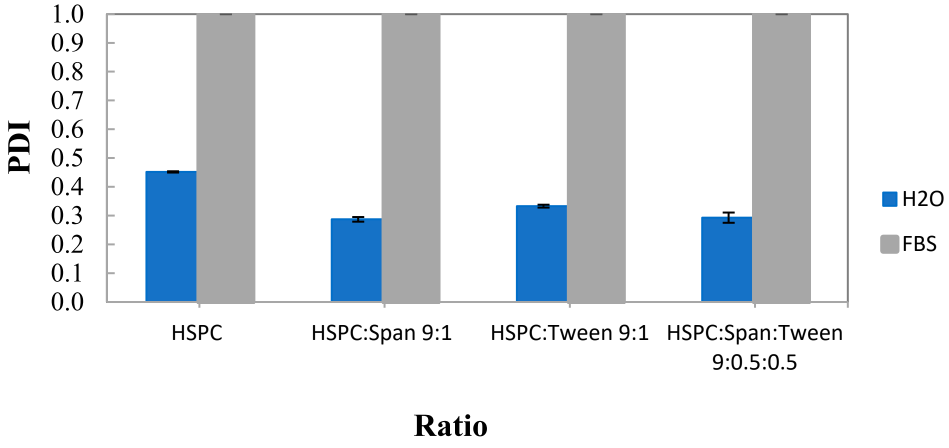

b.

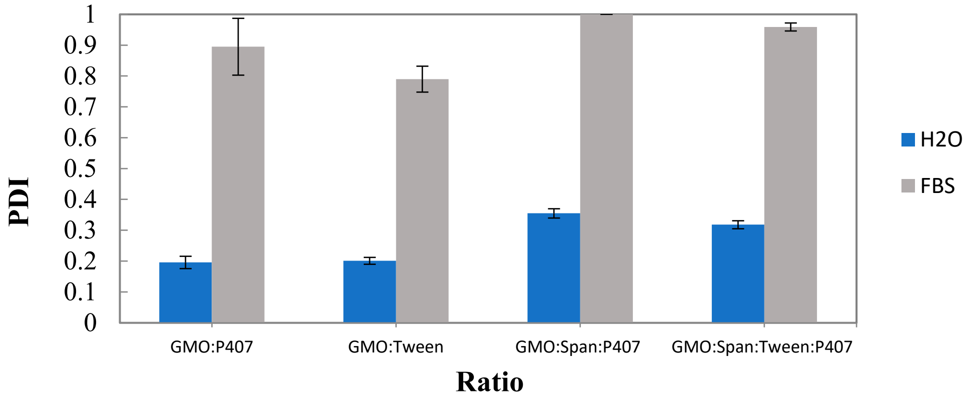

c.

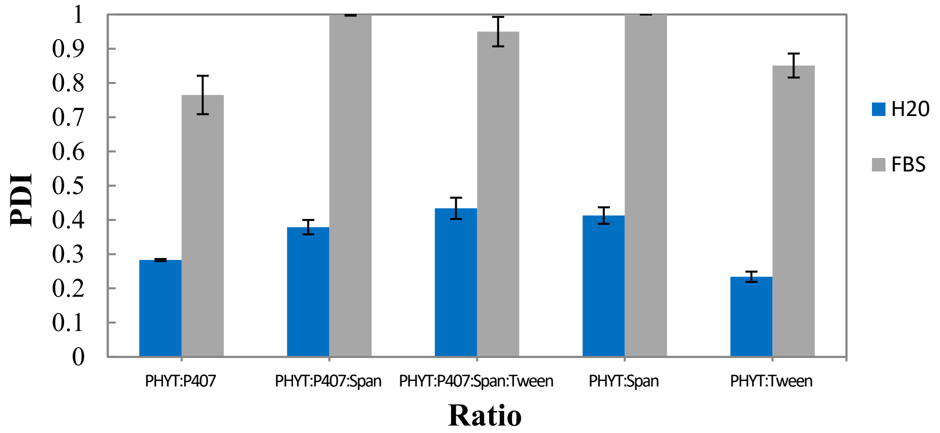

Figure S2. Polydispersity index (PDI) of **a.** HSPC liposomes, **b.** GMO liquid crystalline nanoparticles and **c.** PHYT liquid crystalline nanoparticles in aqueous and biological medium (FBS).

a.

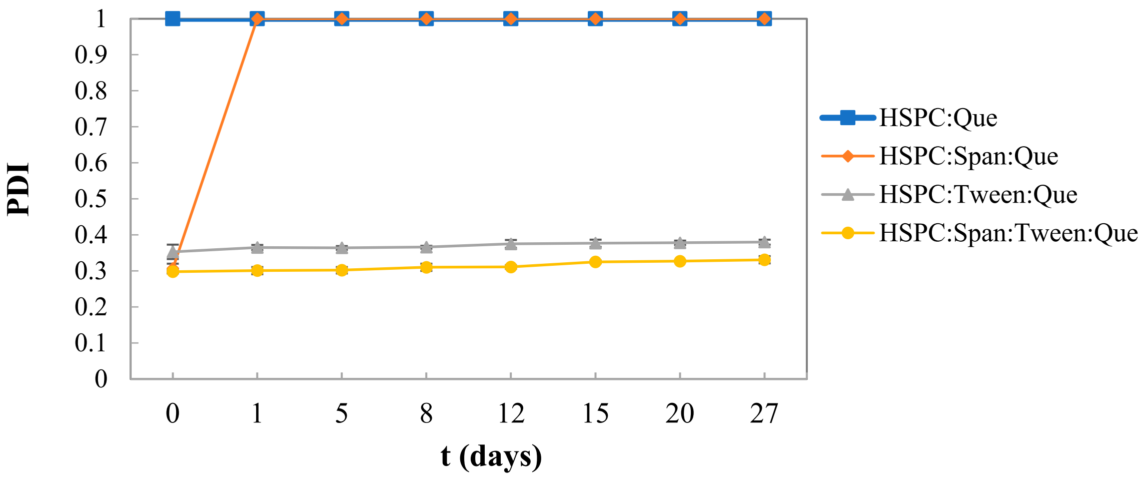

b.

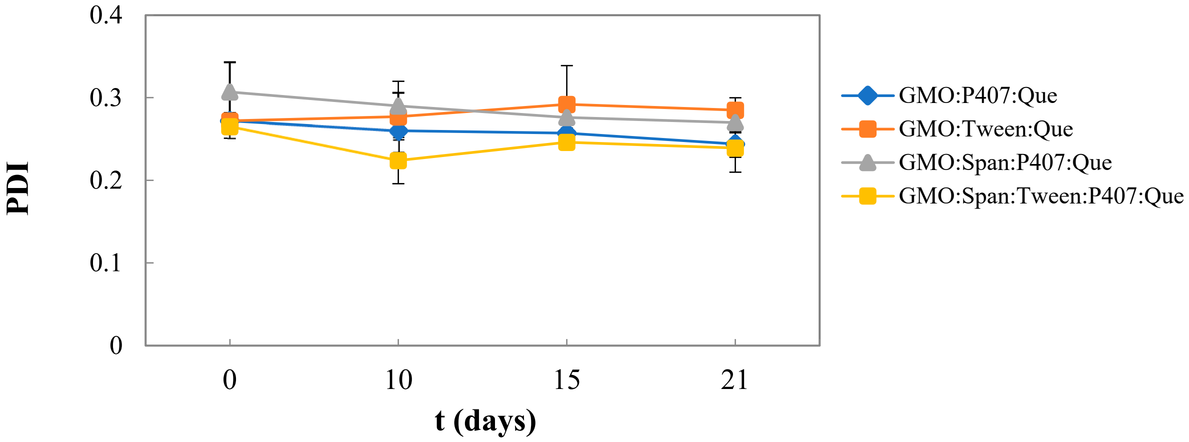

c.

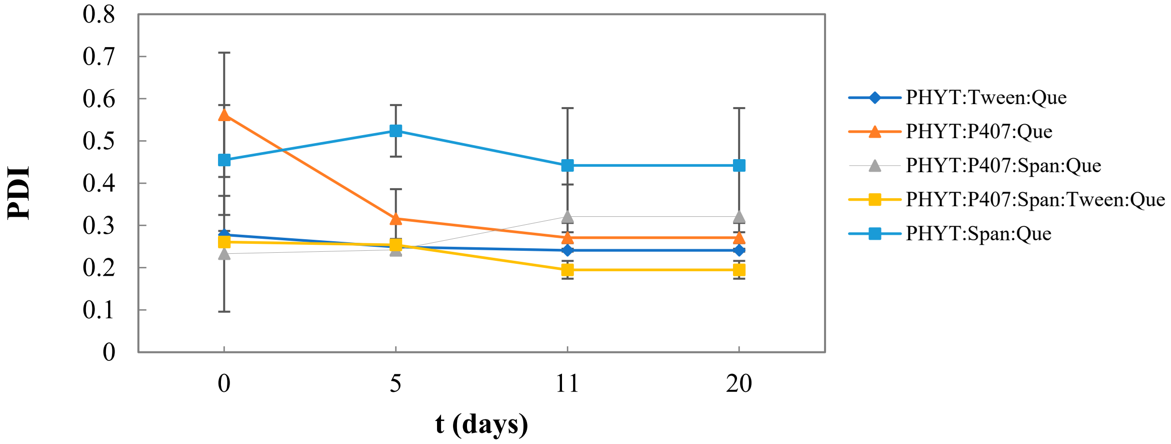

Figure S3. Stability assessment of the polydispersity index (PDI) of **a.** HSPC liposomes, **b.** GMO liquid crystalline nanoparticles and **c.** PHYT liquid crystalline nanoparticles with entrapped Quercetin over time.
